# Effects of Combining Transcranial Direct Current Stimulation with Virtual Reality on Upper Limb Function in Patients with Stroke: A Systematic Review and Meta-Analysis

**DOI:** 10.3390/bioengineering12111205

**Published:** 2025-11-04

**Authors:** Auwal Abdullahi, Thomson W. L. Wong, Shamay S. M. Ng

**Affiliations:** 1 Department of Physiotherapy, Bayero University, Kano 700271, Nigeria; aabdullahi.pth@buk.edu.ng; 2 Department of Rehabilitation Sciences, The Hong Kong Polytechnic University, Hong Kong, China; thomson.wong@polyu.edu.hk

**Keywords:** stroke, transcranial direct current stimulation, virtual reality, activities of daily living, quality of life

## Abstract

**Background:** Persistent upper limb hemiparesis in patients with stroke can result in significant long-term disability and reduced quality of life. Transcranial direct current (tDCS) stimulation and virtual reality (VR) as stand alone or in combination are currently used for the rehabilitation of upper limb function following stroke. **Objectives:** The aim of this study is to determine the effects of combining tDCS with VR on level of motor impairment, motor function, spasticity, ADL, quality of life, manual dexterity, sensation, muscle strength, handgrip strength, cognitive flexibility and speed of processing, motor performance, cognition, and executive function after stroke. **Design:** The study is a systematic review and meta-analysis. **Data Sources and Methods:** PubMED, Embase, Web of Science (WoS), PEDro, and Scopus were searched until June 2023 for randomized controlled trials (RCTs) on the subject matter using the following keywords: stroke, upper extremity, upper limb, virtual reality, virtual rehabilitation, noninvasive brain stimulation, transcranial direct current stimulation, transcortical direct current stimulation, and tDCS. Methodological quality and risks of bias of the included studies were assessed using the PEDro scale and Cochrane risks of bias assessment tool, respectively. Random effect model analysis was used to compute the effect size and standardized mean difference (SMD). **Results:** The results showed that the included studies reported that combining tDCS with VR may improve level of motor impairment, motor function, spasticity, ADL, quality of life, manual dexterity, sensation, muscle strength, handgrip strength, cognitive flexibility and speed of processing, motor performance, cognition, and executive function. However, the result of the meta-analysis showed that it is only superior to the control at improving motor function (SMD = 0.44, 95% CI = 0.09 to 0.79, *p* = 0.01). **Conclusions:** Use of a combination of tDCS with VR may help optimize upper limb function outcomes. However, standardization of the protocol of such an intervention is needed in order to make it applicable in the real world. **Registration:** The study was registered in PROSPERO (registration number, CRD42023435702).

## 1. Introduction

Stroke is a leading cause of disability as a result of impairment in motor, sensory, autonomic, and cognitive functions [1,2,3]. One of the common causes of disability following stroke is persistent upper limb hemiparesis [4]. Persistent hemiparesis of the upper limb can cause activity limitation, since the upper limb is required for carrying out activities of daily living (ADL) such as bathing, cooking, writing, grooming, and washing. The ability to reach, pick, and hold objects using the limb is impaired [5,6]. Inability to carryout ADL can make patients with stroke dependent on others, deny them the right to privacy, and result in reduced quality of life [7,8].

There are various rehabilitation techniques used for upper limb rehabilitation following stroke. However, translating the evidence from these rehabilitation interventions to real world clinical practice in order to provide the desired outcomes still proves difficult [9]. Thus, some scientists are of the opinion that combining two or more of such interventions may maximize outcomes, especially in severe cases [10,11,12,13]. Two of such interventions that are combined are transcranial direct current stimulation (tDCS) and virtual reality (VR) for the rehabilitation of upper limb function [13,14]. Virtual reality provides a real-world-like interactive, multisensory, and fun experience that helps increase the intensity of practice with the upper limb that is required for recovery post stroke [15,16,17,18]. Consequently, use of VR has been reported to improve outcomes such as motor function, spasticity, dexterity, and quality of life [19,20].

Similarly, tDCS has been reported to help enhance recovery of upper limb function following stroke by stimulating neurochemical, neurophysiological, and anatomical changes in the brain, and providing a real-world-like experience [21,22,23,24]. Consequently, use of tDCS has been reported to improve outcomes such as motor function, motor impairment, real-world arm use and, ADL [25]. Thus, combining them together as one intervention may help optimize recovery.

In the above regard, two previous systematic reviews and meta-analyses looked at the effect of combining non-invasive brain stimulation (tDCS and repetitive transcortical magnetic stimulation) or tDCS with VR for upper limb rehabilitation, respectively [26,27]. However, in the first meta-analysis, cross sectional and cross over designs were used [26]. Such designs are susceptible to risks of bias. In addition, a study on transcortical magnetic stimulation (TMS) was included. Unlike tDCS, TMS involves a tedious manpower input and is costly [28]. Thus, use of tDCS seems to be easier and cost-effective.

In the second meta-analysis, the analysis was carried out based on the individual outcome measures—upper extremity Fugl Meyer motor assessment (UEFMA), box and block test (BBT), modified Ashworth scale (MAS), and Barthel index (BI)—not based on the outcomes assessed [27]. In addition, in one of the studies they included, there are two control groups; however, sensitivity analysis was not carried out based on the two control groups. The aim of this review is to carry out a systematic review and meta-analysis of randomized controlled trials (RCTs) with sensitivity analysis to summarize the literature on the effects of combining tDCS with VR on upper limb function in patients with stroke.

## 2. Method

This study is a systematic review and meta-analysis that was conducted according to the Preferred Reporting Items for Systematic Reviews and Meta-Analyses (PRISMA) guidelines [29]. The review was registered in PROSERO (registration number, CRD42023435702), a database for the registration of prospective systematic reviews and meta-analyses.

### 2.1. Criteria Used for Inclusion and Exclusion of Studies

The inclusion criteria followed patients, intervention, comparator or control, and outcomes (PICOs) design. Studies published in the English language were included if they were RCTs involving adult patients with stroke (P) in which use of a combination of tDCS and VR (I) was compared with a control—tDCS, sham tDCS, VR or conventional therapy (C)—on outcomes such as level of motor impairment, motor function, spasticity, ADL, quality of life, manual dexterity, sensation, muscle strength, handgrip strength, cognitive flexibility and speed of processing, motor performance, cognition, and executive function (O).

### 2.2. Literature Search

Five electronic databases, PubMED, Embase, Web of Science (WoS), PEDro, and Scopus, were searched from their inception to October 2025. The keywords used for the search were stroke, upper extremity, upper limb, virtual reality, virtual rehabilitation, noninvasive brain stimulation, transcranial direct current stimulation, transcortical direct current stimulation, and tDCS. The full search strategy is presented in Appendix A. The reference lists of two relevant previous reviews were also searched [27,30].

The search of all the databases was carried out by AA. However, it was verified independently by TWLW.

### 2.3. Selection of Eligible Studies

All the studies provided by the search were exported to Endnote, which was used by AA and TWLW to independently remove duplicates and then select eligible studies for inclusion.

At the beginning, the selection was carried out based on the contents of the title and/or abstracts of the studies. Following that, the remaining studies were selected after reading their full texts. Subsequently, the two researchers held a meeting to agree on their independent selections. The other researcher, SSMN, was contacted if there was any disagreement on a selection. The final results of the search and the selection process are presented using a flowchart.

### 2.4. Extraction of the Data in the Included Studies

The extraction of the data was carried out by AA, although the two other researchers, TWLW and SSMN, verified them to ensure that what was extracted was valid and reliable.

The data that was extracted included characteristics of the participants in the included studies such as mean age, sex, time since stroke, inclusion and exclusion criteria used in the studies, the treatment protocols used in the experimental and control groups including the intensity, mean scores on the outcomes of interest such as level of motor impairment, motor function, spasticity, ADL, quality of life, manual dexterity, sensation, muscle strength, handgrip strength, cognitive flexibility and speed of processing, motor performance, cognition, and executive function.

### 2.5. Risks of Bias and Methodological Quality Assessments of the Included Studies

Two of the researchers, AA and TWLW, independently used Cochrane risks of bias assessment tool and PEDro scale respectively to assess the risks of bias and methodological quality of the included studies.

The Cochrane risks of bias assessment tool is a valid and a reliable tool for the assessment of selection, performance, detection, attrition, and reporting biases, and any other potential bias that may occur when conducting an RCT [31]. The result of the assessment is summarized in a risk of bias graph and a summary table. Similarly, the PEDro scale is an 11-item scale that is used to assess the internal and external validity of RCT [32]. The first item in the scale assesses internal validity, whereas the remaining 10 items assess external validity. In addition, the items that assess external validity are rated on a two-point scale, 0 and 1, which mean a response of no and yes, respectively, to the questions in the items. Thus, total scores on PEDro scale can be regarded as representing low (a score of between 0 and 3), moderate (a score of between 4 and 5), and high (a score of between 6 and 10) methodological quality.

The result of the above assessment is presented in a table. In cases of disagreement following the assessments by the two researchers, the other researcher, SSMN, was consulted for resolution.

### 2.6. Synthesis of the Data from the Included Studies

The extracted data was synthesized using both qualitative and quantitative syntheses. The qualitative synthesis involved a summary of the characteristics of the participants in the included studies such as the intensity of the interventions in the experimental and control groups, number of participants in both groups, the outcomes assessed, and the mean changes post intervention. Following all these, the result was presented using a table.

For the quantitative synthesis, a meta-analysis using a random-effect model was employed to pool together the mean changes in the outcomes of interest to help compare the effects of the experimental and control group interventions. Random-effect model analysis was used because the studies used different outcome measures to measure the same outcomes. Consequently, standardized mean difference was used to compute the effect sizes. In addition, in one of the studies, there are two control groups, tDCS alone and VR alone [33]. Thus, at first, we carried out the meta-analyses with the mean changes on the outcomes of interest for the tDCS alone control group. Following that, we carried out another analysis by substituting the mean changes on the outcomes of interest for the tDCS alone control group with that of the VR alone control group.

Furthermore, the *I*^2^ statistic was used to determine the percentage variation due to heterogeneity between the included studies, and it was only considered to be significant when its value was between 50 and 90% at *p* < 0.05 [34].

The meta-analysis was carried out using RevMan software version 5.4 [35].

### 2.7. How the Evidence Was Interpreted

For the interpretation of the quality of the evidence on the outcomes, GRADE (grading of recommendations, assessment, development, and evaluation) was used [36]. GRADE is an instrument that comprises risks of bias, imprecision, inconsistency, indirectness, and publication bias as domains. Interpretation of the evidence was done based on the seriousness of the risks of bias, imprecision, inconsistency, indirectness, and publication bias in the included studies, the number of included studies, and the sample size in the studies. Finally, the interpretation of the evidence was summarized using a table.

## 3. Results

### 3.1. Narrative Synthesis

#### 3.1.1. Study Selection

**Figure 1:**
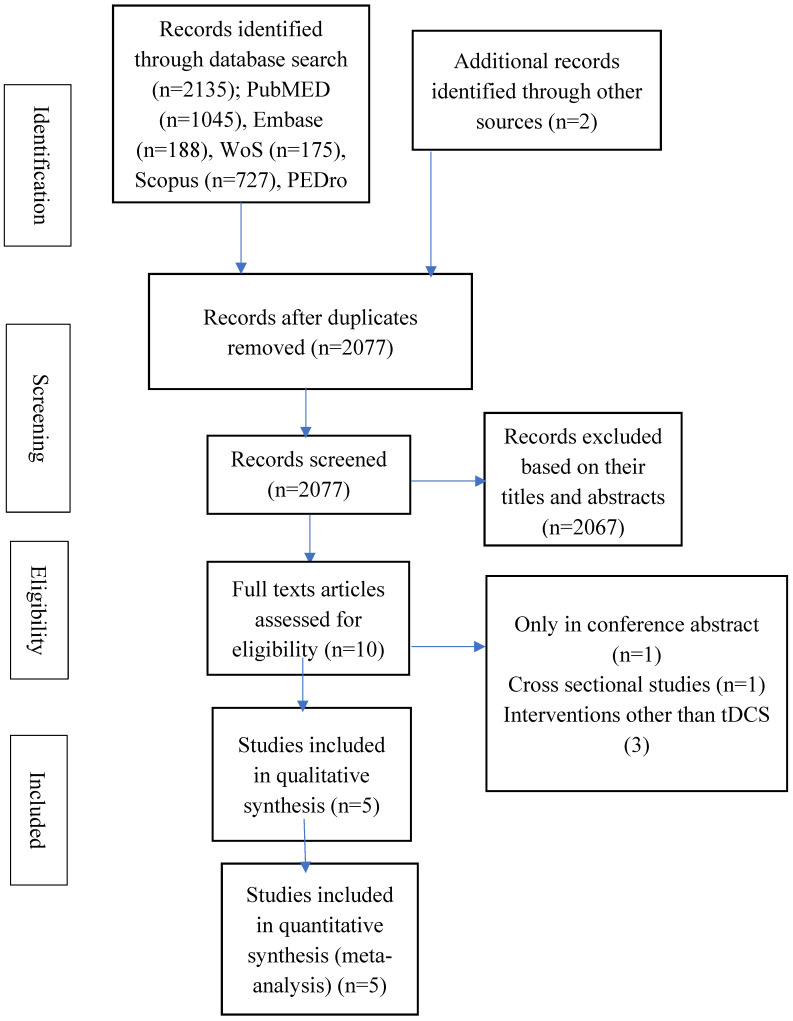
The study flowchart.

The search provided a total of 2137 hits. Out of this number, only five studies were eligible for inclusion in the study [33,37,38,39,40]. In one of the studies, there were two control groups (tDCS alone and VR alone) [33]. Conventional therapy alone was used as a control in one study [39]. All the remaining studies used either tDCS, VR, or sham tDCs with VR as control. See Figure 1 for the flowchart detailing the process of selection of eligible studies.

#### 3.1.2. Characteristics of the Included Studies

The included studies have a total sample size of 168 patients with stroke (range, 20 to 59), a mean age range of 52.3 ± 10.9 to 67.5 ± 6.74 years, and a mean time since stroke range of 16.9 ± 5.5 days to 35 ± 20.3 months. Out of this number, 55 were female and 71 had right sided hemiplegia. The type of stroke the patients had included both ischaemic (*n* = 130) and haemorrhagic stroke (*n* = 38).

In three of the studies, participants with mild or moderate impairment in motor function were included [33,37,40]. In one study, participants had severe impairment in motor function as measured by a Brunnstrom score between I and II and upper extremity Fulg Meyer motor assessment (UEFMA) score of <19 [39]. One study did not provide details on the severity of the impairment in motor function of the included participants [38].

In addition, participants were excluded from the studies if they had a previous history of brain surgery or neurotrauma [32,34,35]; epilepsy or seizure [33,37,38,40]; metallic implant in the brain [33,37,38,39]; severe impairment in cognitive function [33,37,38,39,40]; aphasia [33,38,39]; poor sitting balance [33,39]; severely damaged eyesight [33,39,40]; hemineglect [33,38]; cerebral aneurysm [37]; orthopaedic problems or joint deformity [40]; and pacemakers or artificial cochlea [38,39]. See Table 1 for the details of the characteristics of the included studies.

### 3.2. Methodological Quality and Risks of Bias of the Included Studies

**Figure 2:**
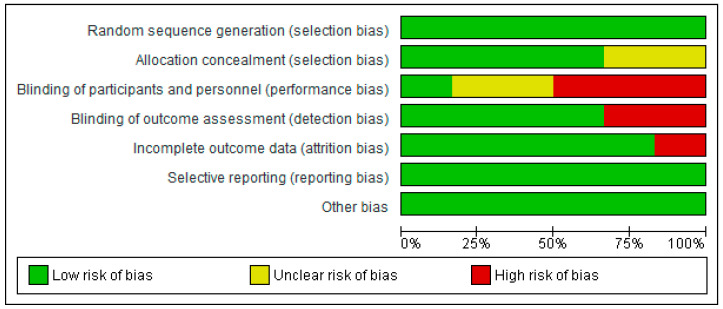
Risk of bias graph.

**Figure 3:**
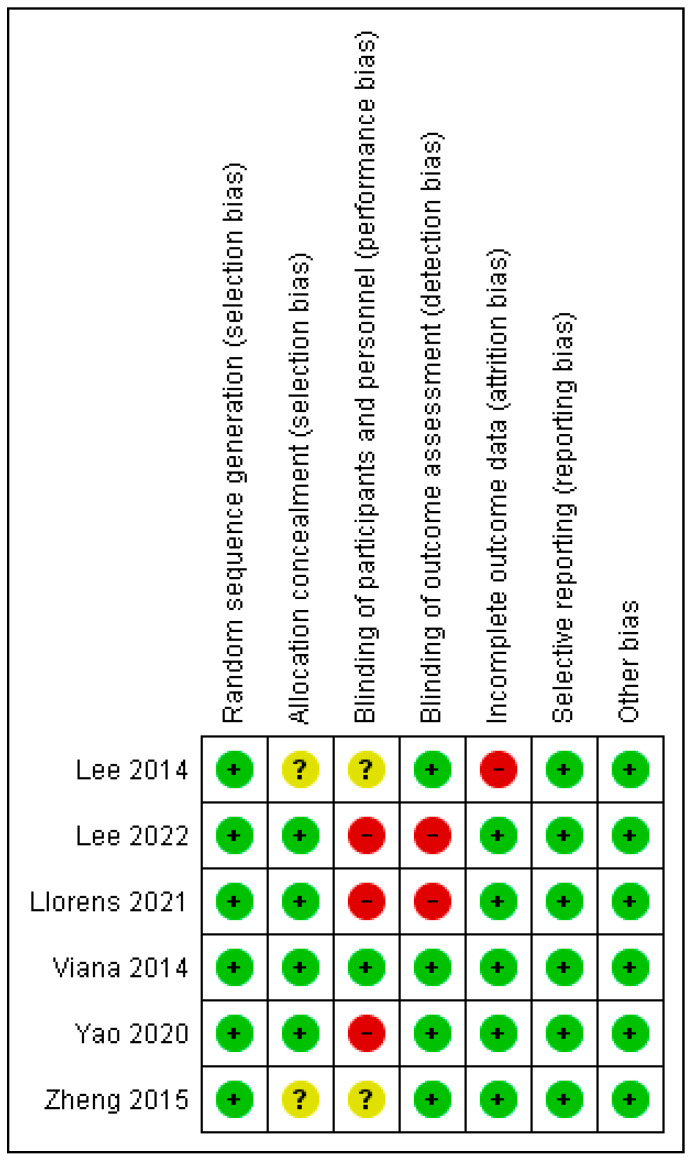
Risks of bias summary table.

All the included studies have high methodological quality. See Table 2 for the methodological quality of the included studies. However, in the studies, there are high risks of bias in blinding of participants and personnel (performance bias) [38,39,40]; incomplete outcome data (attrition bias) [37]; and blinding of outcome assessment (detection bias) [39,40]. In addition, in one of the studies, there were unclear risks of bias in blinding of participants and personnel (performance bias) [33]; and allocation concealment (selection bias) [30]. The risks of bias graph and summary of the included studies are presented in Figure 2 and Figure 3, respectively.

### 3.3. Quantitative Synthesis

#### 3.3.1. Upper Limb Function

**Figure 4:**
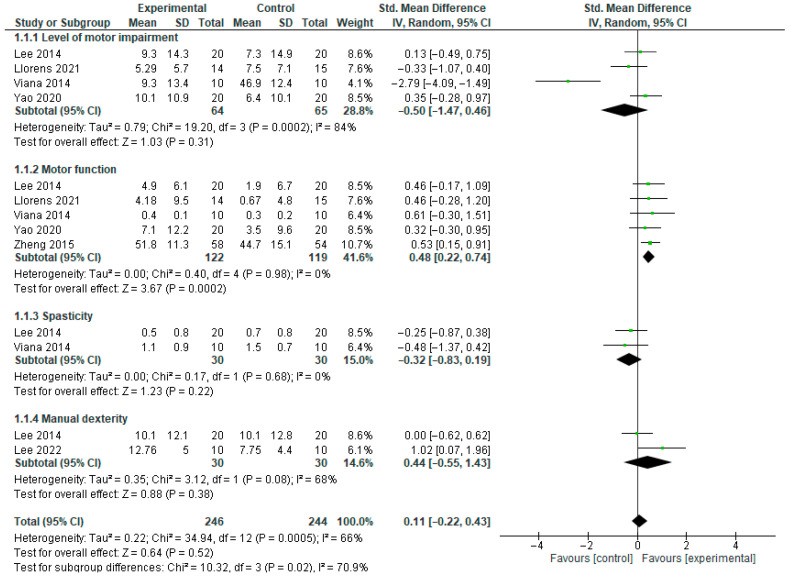
Upper limb function post intervention.

**Figure 5:**
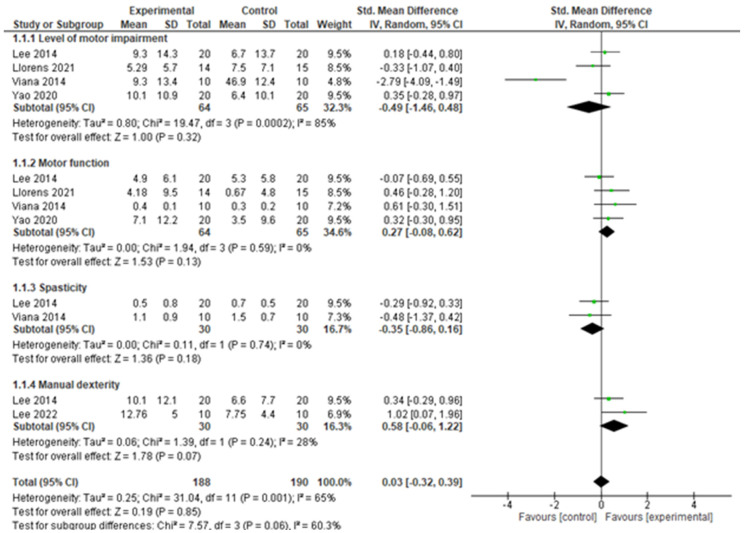
Upper limb function post intervention (sensitivity analysis).

For the level of impairment in motor function, the results showed that there was no significant difference between group (SMD = −0.50, 95% CI = −1.47 to 0.46, *p* = 0.31) post intervention. However, there was a significant heterogeneity between the included studies (*I*^2^ = 84%, *p* = 0.0002). See Figure 4 for the forest plot for this result. Similarly, when sensitivity analysis was carried out by including the values on the outcome of interest when VR was used in place of tDCS as a control in the study by Lee and colleagues [30], the results showed that there is no significant difference between groups (SMD = −0.49, 95% CI = −1.46 to 0.48, *p* = 0.32). In addition, there was a significant heterogeneity between the included studies *I*^2^ = 85%, *p* = 0.0002). See Figure 5 for the forest plot for this result.

For motor function, the results showed that there is a significant difference between groups (SMD = 0.44, 95% CI = 0.09 to 0.79, *p* = 0.01) post intervention. In addition, there is no significant heterogeneity between the included studies (*I*^2^ = 0%, *p* = 0.96). See Figure 4 for the forest plot for this result. When sensitivity analysis was carried out by including the values on the outcome of interest when VR was used in place of tDCS as a control in the study by Lee and colleagues [30]; the result showed that, there is no significant difference between groups (SMD = 0.27, 95% CI = −0.08 to 0.62, *p* = 0.13). However, there was no significant heterogeneity between the included studies *I*^2^ = 0%, *p* = 0.59). See Figure 5 for the forest plot for this result.

For spasticity, the result showed that, there is no significant difference between group (SMD = −0.32, 95% CI = −0.83 to 0.19, *p* = 0.22) post intervention. In addition, there is no significant heterogeneity between the included studies (*I*^2^ = 0%, *p* = 0.68). See Figure 4 for the forest plot for this result. However, when sensitivity analysis was carried out by including the values on the outcome of interest when VR was used in place of tDCS as control in the study by Lee and colleagues [30], the results showed that there is no significant difference between groups (SMD = −0.35, 95% CI = −0.86 to 0.16, *p* = 0.18). However, there was no significant heterogeneity between the included studies *I*^2^ = 0%, *p* = 0.002). See Figure 5 for the forest plot of this result.

For manual dexterity, the results showed that there is no significant difference between groups (SMD = 0.44, 95% CI = −0.55 to 1.43, *p* = 0.38) post intervention. In addition, there is no significant heterogeneity between the included studies (*I*^2^ = 68%, *p* = 0.08). See Figure 4 for the forest plot of this result. However, when sensitivity analysis was carried out by including the values on the outcome of interest when VR was used in place of tDCS as a control in the study by Lee and colleagues [30], the results showed that there is no significant difference between group (SMD = 0.58, 95% CI = −0.06 to 1.22, *p* = 0.07). In addition, there was no significant heterogeneity between the included studies *I*^2^ = 28%, *p* = 0.24). See Figure 5 for the forest plot of this result.

#### 3.3.2. ADL

**Figure 6:**
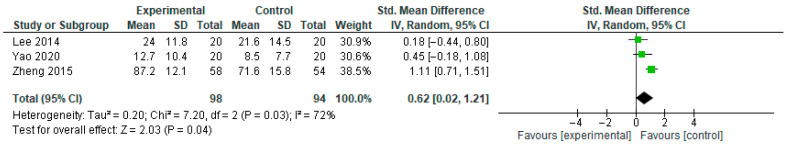
Activities of daily living post intervention.

**Figure 7:**
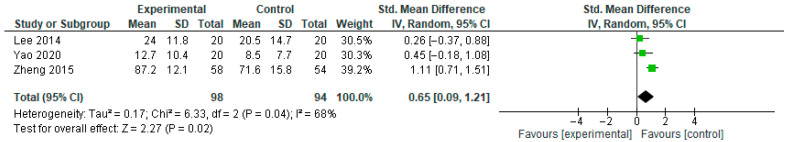
Activities of daily living post intervention (sensitivity analysis).

The results showed that there was no significant difference between groups (SMD = 0.31, 95% CI = −0.13 to 0.75, *p* = 0.17) post intervention. In addition, there was no significant heterogeneity between the included studies (*I*^2^ = 0%, *p* = 0.55). See Figure 6 for the forest plot of this result. Similarly, when sensitivity analysis was carried out by including the values on the outcome of interest when VR was used in place of tDCS as a control in the study by Lee and colleagues [30], the results showed that there was no significant difference between groups (SMD = 0.35, 95% CI = −0.09 to 0.80, *p* = 0.12). In addition, there was no significant heterogeneity between the included studies *I*^2^ = 0%, *p* = 0.67). See Figure 7 for the forest plot detailing the result.

#### 3.3.3. Evidence Quality Interpretation

There seems to be little evidence for an effect of combining tDCS with VR on level of motor impairment and motor function. See Table 3 for more details. On the other hand, based on the effect size for motor function, the value attained minimal clinically important difference on the Wolf Motor Function Test (WMFT) [41]. However, there is heterogeneity in the use of outcome measures for the assessment of motor function between the included studies. This makes it difficult to interpret the findings based on the MCID values. Thus, future studies should standardize the use of outcome measures for evaluating the effects of this intervention.

## 4. Discussion

The aim of this review is to summarize the evidence from randomized controlled trials (RCTs) on the effects of combining tDCS with VR on upper limb function in patients with stroke. From the findings of the individual studies in Table 1, the results show that combining tDCS with VR may improve level of motor impairment, motor function, spasticity, ADL, quality of life, manual dexterity, sensation, muscle strength, handgrip strength, spasticity, cognitive flexibility and speed of processing, motor performance, cognition, and executive function. However, the result of the meta-analysis showed that it is only superior to control at improving motor function. Improvement in motor function is important for patients’ ability to carry out ADL, participation and community reintegration [42,43,44,45]. Ability to carry out ADL, and participation in social, leisure, and religious activities are important for a good quality of life and returning to work following stroke [46,47,48].

On the other hand, the meta-analysis did not show any significant difference between the experimental and the control group in level of motor impairment, ADL, manual dexterity, and spasticity post intervention. This could be due to several factors. Firstly, the control interventions in the included studies were mostly tDCS and VR. These interventions have been shown to individually improve upper limb function outcomes in patients with stroke [15,16,17,18,21]. Secondly, all the included studies have relatively low sample sizes. Studies with low sample sizes are more unlikely to detect significant difference between groups [49,50].

In addition, the types of outcomes assessed and the outcome measures used in the studies could also play a significant role in the outcomes observed. For instance, measures that are considered suitable in every stroke trial were only used for upper limb motor function [51]. These measures are UEFMA and action research arm test (ARAT). Similarly, another limitation of the included studies is the lack of standardization of the experimental protocols. For instance, in one of the studies, the duration of the intervention was 3–5 times a week, which seems to mean some patients received the interventions 2, 4, or 5 times a week [39]. This can make reproducibility of the trials and their applications in real-world practice very difficult.

Nevertheless, combining more than one intervention has been said to be a safe and applicable intervention for upper limb function rehabilitation in stroke [52]. Consequently, in recent times, researchers have advocated the use of such an intervention [10,11,12,13]. In particular, in the case of combining tDCS with VR, the former helps to modulates the nervous system, the latter [24], the latter provides a real-world-like environment for patients to engage with rehabilitation to optimize outcomes [18]. Thus, the finding of this study can help guide practice and future research.

### Limitations of the Study

Although this study has several strengths, such as an extensive search of the literature, the use of meta-analysis, and interpretation of the evidence using a valid and reliable method, it also has some limitations. One of the limitations is the presence of heterogeneity in terms of inclusion of studies with heterogenous control groups (clinical heterogeneity), and inconsistency between studies in the use of outcome measures (statistical heterogeneity). Heterogeneity can negatively affect the reliability of the findings of studies [53]. Secondly, inclusion of studies that are published only in the English language is another limitation that limits potential quality studies.

## 5. Conclusions

Use of a combination of tDCS with VR may help optimize upper limb function outcomes. However, standardization of the protocol of such an intervention is needed in order to make it applicable in the real world. Therefore, more studies with standardized protocols are needed to provide a more valid and reliable evidence. In particular, the studies should use adequate sample sizes in order to help prevent type II error. In addition, the studies should include outcome measures such as the motor activity log (MAL) which assess real-world upper limb use in order to help the full spectrum of upper limb recovery post stroke. Furthermore, the studies need to assess the long-term effects of combining tDCS with VR.

## Figures and Tables

**Table 1:** Characteristics of the included studies.

References	*N*	Stroke Duration	Mean Age (Years)	Intervention	Outcomes	Findings	Adverse Events
Lee et al. [33]	*N* = 59; experimental (*n* = 20, females = 8); control 1 (*n* = 19, females = 9); control 2 (*n* = 20, females = 11).	Experimental = 17.8 ± 7.3 days; control 1 = 17.4 ± 9.4 days; control 2 = 16.9 ± 5.5 days.	Experimental = 63.1 ± 10.3; control 1 = 60.3 ± 11.3; control 2 = 60.6 ± 14.1.	Experimental = received tDCs (2 mA for 20 min) during virtual reality therapy. Control 1 = received tDCs (2 mA for 20 min) during occupational therapy Control 2 = received virtual reality therapy instead of occupational therapy. The interventions in all the groups were carried out for 30 min per day, 5 times a week for 3 weeks. In addition, participants in all the groups received conventional rehabilitation of the same intensity and time.	Spasticity (MAS), muscle strength (MMT), manual function (MFT), level of motor impairment (UEFMA), manual dexterity (BBT), and activities of daily living (MBI).	Muscle strength, manual function, level of motor impairment and activities of daily living improved post intervention in all groups. However, manual function and level of motor impairment improved significantly in the experimental group compared to the two other groups.	No major adverse events.
Viana et al. [37]	*N* = 20 experimental (*n* = 10, females = 1); control (*n* = 10, females = 3).	Experimental = 31.9 ± 18.2 months; control = 35 ± 20.3 months.	Experimental = 56.0 ± 10.2; control = 55.0 ± 12.2.	Experimental = received 1-h virtual reality (VR) therapy and 13 min of 2 mA tDCs before the VR therapy per day, 3 times a week for 5 weeks. Control = received VR with the same intensity as in the experimental group, and sham tDCs for the same period.	Level of motor impairment (UEFMA), motor function (WMFT), spasticity (MAS), handgrip strength (hand-held dynamometry), and quality of life (SSQoLQ).	All outcomes improved in both groups post intervention. However, spasticity improved significantly higher in the experimental group compared to the control.	No adverse events reported.
Yao et al. [38]	*N* = 40 experimental (*n* = 20, females = 6); control (*n* = 20, females = 3).	Experimental = 60.5 ± 35.5 days; control = 56.5 ± 33.3 days.	Experimental = 63.0 ± 7.5; control = 66.2 ± 6.2.	Experimental = received a simultaneous 20 min virtual reality (VR) therapy and tDCs (2 mA) per day, 5 times a week for 2 weeks. Control = received VR and sham tDCs for the same period.	Adverse events (adverse effect of tDCs questionnaire), level of motor impairment (UEFMA), motor function (ARAT), activities of daily living (BI).	All outcomes improved in both groups post intervention. However, the improvement in all the outcomes was significantly higher in the experimental group compared to the control.	Tingling and itching sensations lasting between 1 and 2 min.
Llorens et al. [39]	*N* = 29 experimental (*n* = 14, females = 3); control (*n* = 15, females = 4).	Experimental = 8.7 ± 2.3 months; control = 9.3 ± 2.4 months.	Experimental = 57.6 ± 6.9; control = 52.3 ± 10.9.	Experimental = received a simultaneous 30 min virtual reality (VR) therapy and tCDs (2 mA) in addition to 30 min conventional therapy per day, 3–5 times a week for 5 weeks. Control = received 30 min conventional therapy per day, 3–5 times a week for 5 weeks.	Level of motor impairment (UEFMA), sensation (NSA) and motor function (WMFT).	All outcomes improved in both groups post intervention. However, the improvement in level of motor impairment and motor function were significantly higher in the experimental group compared to the control.	Not reported.
Lee et al. [40]	*N* = 20; experimental (*n* = 10, females = 4); control (*n* = 10, females = 3).	Experimental = 3.75 ± 1.48 months; control = 4.12 ± 1.55 months.	Experimental = 67.5 ± 6.74; control = 65.00 ± 5.73.	Experimental = received tDCs (2 mA for 20 min) during virtual reality therapy, 5 times a week for 4 weeks Control 1 = received sham tDCs (2 mA for 20 min) during virtual reality therapy, 5 times a week for 4 weeks.	Manual dexterity (BBT), use of the hand in activities of daily living (JTHFT), selective attention and cognitive flexibility (ST), and speed of processing, motor performance, cognition, and executive function (TMT).	All outcomes improved in both groups post intervention. However, manually dexterity and selective attention and cognitive flexibility improved significantly higher in the experimental group.	Not reported.

Key: tDCs = transcortical direct stimulation, rMT = resting motor threshold, MAS = modified Ashworth scale, MMT = manual muscle test, MFT = manual function test, UEFMA = upper extremity Fugl Meyer motor assessment, BBT = box and block test, MBI = modified Barthel index, WMFT = Wolf motor function test, SSQoLQ = stroke specific quality of life questionnaire, ARAT = action research arm test, BI = Barthel index, NSA = Nottingham sensory assessment. Keywords: BBT = box and block test, JTHFT = Jebsen-Taylor Hand Function Test, ST = The stroop test, TMT = The trail making test.

**Table 2:** Methodological quality of the included studies.

Study	Eligibility Criteria Specified	Random Allocation	Concealed Allocation	Comparable Subjects	Blind Subjects	Blind Therapists	Blind Assessors	Adequate Follow-Up	Intention to Treat Analysis	Between Group Comparison	Point Estimation and Variability	Total Score
Lee et al. [33]	Yes	1	1	1	0	1	1	0	0	1	1	7/10
Viana et al. [37]	Yes	1	1	1	1	1	1	1	1	1	1	10/10
Yao et al. [38]	Yes	1	1	1	1	0	1	0	1	1	1	8/10
Llorens et al. [39]	Yes	1	1	1	0	0	0	1	1	1	1	7/10
Lee et al. [40]	Yes	1	1	1	0	0	0	1	1	1	1	7/10

**Table 3:** Evidence quality assessment.

						Number of Participants			
Outcome	Number of studies	Risks of bias	Inconsistency	Indirectness	Imprecision	Experimental	control	Effect size (95% CI)	Overall certainty of the evidence
Level of motor impairment	4	Not serious	Not serious	Not serious	Serious ^b^	64	65	−0.50 (−1.47 to 0.46)	⨁⨁◯◯ Low
Motor function	4	Not serious	Not serious	Not serious	Serious ^b^	64	65	0.44 (0.09 to 0.79)	⨁⨁◯◯ Low
Spasticity	4	Not serious	Very serious ^a^	Not serious	Serious ^b^	30	30	−0.32 (−0.83 to 0.19)	⨁⨁◯◯ Low
Manual dexterity	2	Not serious	Very serious ^a^	Not serious	Serious ^b^	30	30	0.44 (−0.55 to 1.43)	⨁◯◯◯ Very low
Activities of daily living	2	Not serious	Very serious ^a^	Not serious	Serious ^b^	40	40	0.31 (−0.13 to 0.75)	⨁◯◯◯ Very low

^a^ Significant heterogeneity ^b^ Sample size < 400.

## Data Availability

All the data for this study is included within the manuscript.

## Appendix A

The search strategy used in all the databases

PubMED

((((((((Stroke) AND (Upper extremity)) OR (Upper limb)) AND (Virtual reality)) OR (Virtual rehabilitation)) AND (Noninvasive brain stimulation)) OR (Transcranial direct current stimulation)) OR (Transcortical direct current stimulation)) OR (tDCS)

Embase

((((‘stroke’/exp OR stroke) AND upper AND extremity OR (upper AND limb)) AND virtual AND reality OR (virtual AND rehabilitation)) AND noninvasive AND brain AND stimulation OR (transcranial AND direct AND current AND stimulation) OR (transcortical AND direct AND current AND stimulation) OR tdcs) AND [randomized controlled trial]/lim

Scopus

(ALL (stroke) AND ALL (upper AND extremity) OR ALL (upper AND limb) AND ALL (virtual AND reality) OR ALL (virtual AND rehabilitation) AND ALL (noninvasive AND brain AND stimulation) OR ALL (transcranial AND direct AND current AND stimulation) OR ALL (transcortical AND direct AND current AND stimulation) OR ALL (tdcs)) AND (LIMIT-TO (DOCTYPE, “ar”)) AND (LIMIT-TO (LANGUAGE, “English”))

WoS

((((((((Stroke) AND (Upper extremity)) OR (Upper limb)) AND (Virtual reality)) OR (Virtual rehabilitation)) AND (Noninvasive brain stimulation)) OR (Transcranial direct current stimulation)) OR (Transcortical direct current stimulation)) OR (tDCS)

PEDro

Stroke AND virtual reality

Stroke AND transcranial direct current stimulation
